# Editorial: Use of Smartphone Applications to Increase Physical Activity and Fitness

**DOI:** 10.3389/fpubh.2021.713306

**Published:** 2022-01-04

**Authors:** Robertas Damaševičius, Jayoung Kim, Victor Z. Dourado

**Affiliations:** ^1^Faculty of Applied Mathematics, Silesian University of Technology, Gliwice, Poland; ^2^Cedars Sinai Medical Center, Los Angeles, CA, United States; ^3^Department of Human Movement Sciences, Federal University of São Paulo, São Paulo, Brazil

**Keywords:** smartphone services, physical activity, persuasive technologies, healthy lifestyle, digital apps, e-health

The benefits of physical activity to the impediment and remedy of many illnesses are evident. For example, daily exercising prevents hypertension, reduces the risk of obesity, and improves mental health and quality of life. Yet, despite its consistent health benefits, one in four adults and three in four adolescents still do not follow the minimum physical activity of at least 150 min of moderate to vigorous physical activity a week as recommended by the World Health Organization. The current guidelines are “every step count,” i.e., “doing some physical activity is better than doing none.” Doing any kind of physical activity will benefit adult's health. Adults should start with small amounts of physical activity and gradually increase frequency, intensity, and duration once they feel prepared. In this context, technology can play an essential role in breaking sedentary behavior.

Recent technological advances such as smartphones and their apps have been released to interact and evaluate human bodily activity. Recent evidence indicates that app-based interventions for physical activity have a small-to-moderate positive effect on physical activity measures, corresponding to 1,850 steps per day. Although effective in promoting a more physically active lifestyle, more extended studies are needed to assess the impact of different intervention components on long-term engagement and effectiveness.

The deployment of apps, primarily based on behavioral exchange methods, would increase cognitive patterns encouraging exercise and physical activity. For that reason, the critical elements ought to be gamification and personalization.

Despite their increasing use, the smartphone apps for a healthy lifestyle and physical monitoring still lack a scientific background. Among multiple fitness and health apps, only some are primarily based on persuasive techniques. Advanced functions such as data mining and machine learning for personalizing bodily exercises are scarce. The most crucial difficulty of the handy apps, with or except sport elements, is to support long-term motivation to keep exercising. Unfortunately, smartphone applications for physical activity offer only eight (1) behavior change techniques among at least 93 (2).

The articles published in this Research Topic evaluated the consequences of services provided by smartphone apps based on motivational and gamification theories, discussed personalization of eHealth monitoring, focusing on the personalization approach by way of combining data acquisition from smartphones, wearables, and sensors, and discussed the determinants of long-term use of smartphone apps for a healthy lifestyle.

Oyibo and Vassileva present a study using the Persuasive Technology Acceptance Model (PTAM) for fitness software directed at motivating bodily exercise at home using Canada and Nigeria as case study countries. The study looked at the biggest influences on users' intention to use a fitness app (perceived usefulness, perceived usability, perceived aesthetics, and perceived credibility); the moderating impact of culture; and how perceived persuasiveness mediates the direct effect of perceived usefulness on the intention to use a health app.

Wattanapisit et al. present virtual run programs that encourage individuals to run with a challenge and use an online platform to report the running activities. The proof supports the exciting outcomes of using a mixture of behavioral challenges and physical activity trackers on fitness outcomes. Finally, the article debates the intent and management of virtual run programs for promoting physical activity.

Nibbeling et al. describe a smartphone-based Playful Active data-driven Urban Living (PAUL) application developed to encourage wholesome inactive city residents to come to be extra physically active in public city spaces. The persuasive techniques protected in the app are derived from health behavior change theories. The study analyses the differences in preferences between people, displaying a want for an app personalized and adapted primarily based on these personal preferences.

Sporrel et al. explore whether location-specific facts on performing physical activity in the constructed surroundings are an enhancement to a physical activity app. The results of this research knowledgeable the graph of a personalized cellular fitness (mHealth) application for running, walking, and performing strength exercises. The PAUL application has been developed by a consortium including Universities in The Nederland and Brazil. It intends to include a consistent combination of a more significant number of behavior change techniques, gamification, data mining, social network, and interaction between the users and the natural and built environment.

Finally, Aldenaini et al. present a systematic review of the performance of mobile phone-based persuasive technology in advertising physical activity while discouraging sedentary behavior. Based on the results of 80 studies, the article analyses the trends in smartphone-based persuasive technologies in physical activity and sedentary behavior, inclusive of smart technologies applied with the mobile phone, behavioral theories, and persuasive techniques. Finally, the study discusses the contributions and limitations of current cellular phone-based persuasive technology interventions to increase motivation for physical activity and discourage sedentary behavior and presented guidelines that will direct future research.

Briefly, the current issue of Frontiers in Public Health has shown that smartphone apps for physical activity and fitness hold promise for propping individuals into more physically active behavior and encouraging regular exercise. However, it was also clear that the technologies developed for this purpose so far lack a scientific basis. Moreover, the apps lack behavior change techniques and do not take advantage of the advancement in data mining and artificial intelligence to make the experience of app users more personalized. Finally, it is noteworthy here that the effects of smartphone apps for physical activity have been evaluated in non-adequate research designs, i.e., conventional randomized clinical trials. Therefore, Sequential Multiple Assignment Randomized Trials (SMART) and micro-randomized clinical trials for Just-in-Time interventions may be capable of keeping up with the dynamism of technology. These strategies are essential to consolidate mHealth as a consensual tool to improve the level of physical activity and fitness and break sedentary behavior.

## Author Contributions

RD contributed to the writing of this editorial. All authors contributed to the article and approved the submitted version.

## Funding

This work was funded by São Paulo Research Foundation (FAPESP) grant number 2016/50249-3.

## Conflict of Interest

The authors declare that the research was conducted in the absence of any commercial or financial relationships that could be construed as a potential conflict of interest.

## Publisher's Note

All claims expressed in this article are solely those of the authors and do not necessarily represent those of their affiliated organizations, or those of the publisher, the editors and the reviewers. Any product that may be evaluated in this article, or claim that may be made by its manufacturer, is not guaranteed or endorsed by the publisher.
